# Pudendal Neuralgia: Two case reports with laparoscopic nerve decompression

**DOI:** 10.52054/FVVO.15.2.073

**Published:** 2023-06-30

**Authors:** N Habib, G Centini, J.S. Klebanoff, R Fernandes, M Giorgi, G.N. Moawad, J Bakar

**Affiliations:** Department of Obstetrics and Gynaecology, Francois Quesnay Hospital, Mantes-La-Jolie 78201, France; Department of Molecular and Developmental Medicine, Obstetrics and Gynaecological Clinic, University of Siena, Siena 53100, Italy; Department of Obstetrics and Gynaecology, Main Line Health System, Wynnewood 19096, United Sates of America; Discipline of Gynaecology, Department of OBGYN, Instituto do Câncer do Estado de São Paulo ICESP, FMUSP, Sao Paulo 01246-000, Brazil; Gynaecology Department, George Washington University School of Medicine and Health Sciences, Washington 20052, United Sates of America

**Keywords:** Pudendal nerve, neuralgia, neurolysis, pelvic pain, laparoscopy

## Abstract

Pudendal neuralgia (PN) is a rare and underestimated condition. The reported incidence by the International Pudendal Neuropathy Association is 1/100000. However, the actual rate may be significantly higher, with a propensity for women. It is most frequently caused by an entrapment of the nerve at the level of the sacrospinous and sacrotuberous ligament, also known as pudendal nerve entrapment syndrome. Due to the late diagnosis and inadequate management, pudendal nerve entrapment syndrome often leads to considerable reduction in the quality of life and high health care costs. The diagnosis is made using Nantes Criteria, in conjunction with the patient’s clinical history and physical findings. Clinical examination with an accurate assessment of the territory of the neuropathic pain is mandatory to set the therapeutic strategy. The aim of the treatment is to control the symptoms and it usually starts with conservative approaches which include analgesics, anticonvulsants, and muscle relaxants. Surgical nerve decompression can be proposed after failure of conservative management. The laparoscopic approach is a feasible and appropriate technique to explore and decompress the pudendal nerve, and to rule out other pelvic conditions that can cause similar symptomatology. In this paper, the clinical history of two patients affected by compressive PN is reported. Both patients underwent laparoscopic pudendal neurolysis suggesting that the treatment for PN should be individualised and carried out by a multidisciplinary team. When conservative treatment fails, laparoscopic nerve exploration and decompression is an adequate option to propose and should be performed by a trained surgeon.

## Introduction

Pudendal neuralgia (PN) is defined as a chronic, severe and disabling pain or discomfort, affecting the pelvis and the genitals, due to a direct lesion or entrapment of the pudendal nerve anywhere along its course. As estimated by the International Pudendal Neuropathy Foundation, the incidence of this condition is 1 per 100000, but the actual incidence may be higher than reported (Hibner et al., 2010). The pudendal nerve is a pelvic somatic nerve emerging from fibres of the second, third and fourth ventral rami of the sacral roots on both sides of the pelvis. The nerve descends caudally and posteriorly between the lower edge of the piriformis muscle, coursing behind the coccygeus muscle, and leaves the pelvis through the greater sciatic foramen. Once outside the pelvis, it travels between the sacrospinous and the sacrotuberous ligament, accompanied by the pudendal vessels. It enters the pelvis through the lesser sciatic foramen to continue into the Alcock’s canal. In women, the distal portion of the pudendal nerve divides into three terminal branches: the dorsal nerve of the clitoris, the perineal nerve that gives the posterior labia nerve, and the inferior rectal nerve that gives the rectal plexus. The first and most anterior branch innervates the genitalia working as a sensory branch to the clitoris; the intermediary branch descends into the perineal pouch with sensory fibres to the urogenital triangle and the posterior portion of the labia majora, and motor fibres innervating the perineal pouch; the rectal branch is responsible for the motor innervation of the levator ani and external anal sphincter muscles (Montoya et al., 2011; Maldonado et al., 2015).

Often under diagnosed and mismanaged, PN can lead to severe sequelae with considerable quality of life impairment (Hibner et al., 2010; Ploteau et al., 2015). Although damage to the pudendal nerve can occur anywhere along its course, it is most frequently due to an entrapment at the level of the sacrospinous and the sacrotuberous ligaments (Robert et al., 1998; Filler, 2009), with a suggestive symptomatology known as pudendal nerve entrapment syndrome (Labat et al., 2008). The symptoms are often consistent with neuropathic pain, characterised by a feeling of numbness, burning, tingling and occasionally a sensation of electrical impulse. The clinical presentation can emerge progressively or appear instantaneously and may worsen when sitting and improve on standing. Hypersensitisation and irritation can also occur due to inflammation especially when an infection or endometriosis is in close proximity to the nerve (Ramsden et al., 2003). Iatrogenic damage can occur during prolapse repair, mesh insertion, urinary incontinence surgery or after vaginal delivery (Sultan et al., 1994; Lien et al., 2005; Possover and Lemos, 2011; Marcus- Braun et al., 2012; Sancak et al., 2016). As stated by Labat et al. (2008) the diagnosis of compressive PN is primarily clinical using Nantes criteria. Essential criteria for diagnosis include pain in the territory of the pudendal nerve (from the anus to the clitoris), pain predominantly experienced while sitting, pain that does not wake the patient at night, pain with no objective sensory impairment and pain relieved by diagnostic pudendal nerve block. Complementary diagnostic criteria include burning, shooting, stabbing pain, numbness, allodynia or hyperpathia, rectal or vaginal foreign body sensation (sympathalgia), worsening of pain during the day, predominantly unilateral pain, pain triggered by defecation, presence of exquisite tenderness on palpation of the ischial spine and clinical neurophysiology findings in men or nulliparous women.

The treatment should be selected according to the aetiology of PN, combining ways to relieve pain including analgesics, anticonvulsants, and muscle relaxants. When conservative measures are ineffective, surgical neurolysis must be considered.

In any case, treatment should not be delayed: the earlier, the more efficient.

This case report details the clinical histories of two patients with PN. The first patient had idiopathic PN due to unexplained nerve compression between the sacrospinous and the sacrotuberous ligaments; the second patient showed an iatrogenic form of the disease following mesh insertion for stress urinary incontinence.

## Surgical technique

Laparoscopic transperitoneal pudendal neurolysis was performed in both cases. Here is the description of the steps followed.

**Figure 1:**
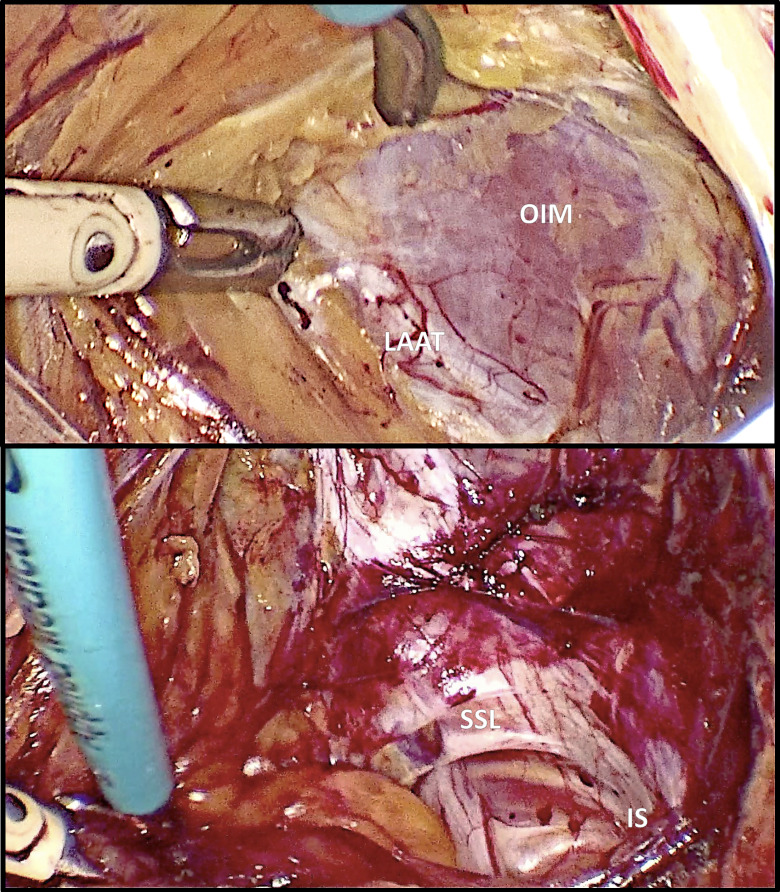
The ventral (above) and dorsal (below) landmarks essential for the preliminary surgical steps for the access to the area of the pudendal nerve. Obturator internus muscle OIM; Levator ani arcus tendinous LAAT; Ischial spine IS; Sacrospinous ligament SSL.

The surgery starts with an exploration of the peritoneal cavity, ruling out endometriosis lesions in the upper abdomen and the pelvis, followed by bilateral or side-specific pudendal neurolysis. Pudendal neurolysis consists in the development of the paravesical space laterally to the obliterated umbilical artery until the round ligament cranially, the external iliac vessels, the obturator structures laterally and the levator ani muscle deeply. The arcus tendinous of the endopelvic fascia is identified and followed until the ischial spine (Figure 1). The coccygeus muscle fibres and the sacrospinous ligament are coagulated and transected whilst protecting the underneath structures. This step allows the decompression and the mobilisation of the pudendal nerve until its entry in the Alcock’s canal.

### Case 1

A 23-years old nulliparous patient complained of chronic perineal pain for two years. Symptoms worsened in the seated position, with numbness of the clitoris and burning in the right labia majora.

In addition, she suffered from dysmenorrhea rated 7 out of 10 using a visual analogue scale (VAS), and severe dyspareunia (VAS score: 8/10). She denied either urinary or defecation symptoms. She had been treated with pain medication, antiepileptic drugs, and physical therapy without improvement for 10 months. Therefore, she had a diagnostic laparoscopy showing no clear explanation of her pain and no signs of endometriosis.

On clinical examination, palpation of the right sacrospinous ligament triggered the pain, and a right pudendal nerve block was administered leading to temporary improvement of her symptoms. The pudendal block was performed transvaginally at a dose of 4.5 mg/kg. It was repeated two other times adding corticoids (Diprostene 1 ml) resulting in pain relief for two and three months after the injection, respectively. A diagnosis of pudendal nerve entrapment between the sacrospinous and the sacrotuberous ligaments was made. However, the clinician’s recommendation of transgluteal pudendal neurolysis was refused by the patient. Thus, she had a consultation in Francois Quesnay Hospital and a laparoscopic pudendal neurolysis was suggested and desired by the patient.

**Figure 2:**
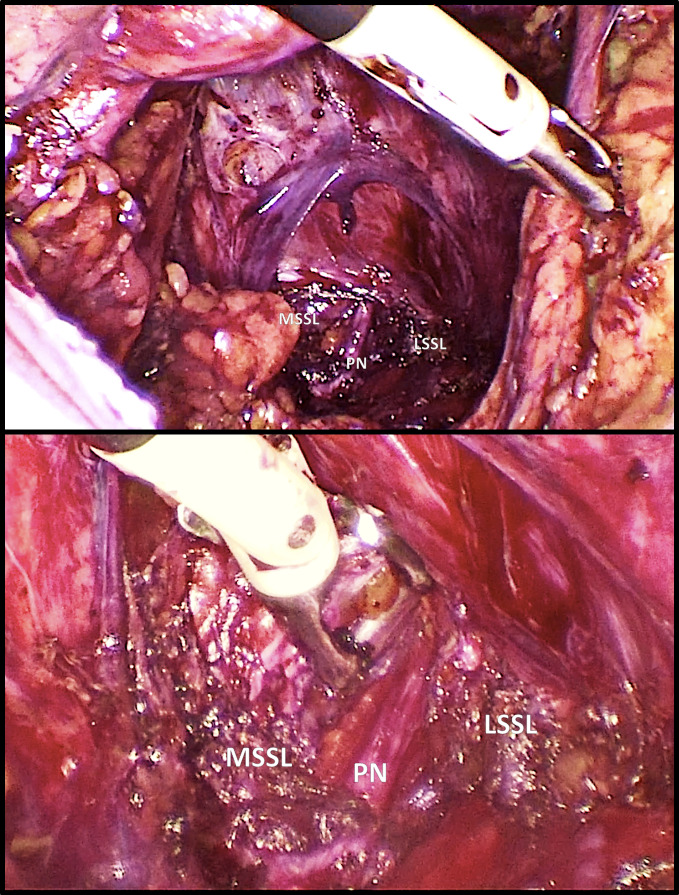
Case 1 laparoscopy. The pudendal nerve is visible after the transection of the sacrospinous ligament with a general overview (above) and in details (below). Pudendal nerve PN; Cut edges of the sacrospinous ligament: medial part MSSL, and lateral part LSSL.

The surgery was performed successfully. The pudendal nerve was medialised because of the compression between the sacrospinous and the sacrotuberous ligament. The decompression of the nerve was achieved through the section of the sacrospinous ligament and its release until the entrance into the Alcock’s canal (Figure 2). Barrier gel was injected around the nerve to decrease the possibility of nearby tissues adhesions.

The postoperative follow-up was uneventful, and the patient was discharged one day after the surgery. The patient was revaluated at three and six months after the surgery. She referred a considerable improvement of her symptomatology and quality of life. She reported minor dyspareunia (VAS score: 4/10) and dysmenorrhea (VAS score: 3/10), managed with occasional pain killers. No other symptoms were described.

### Case 2

49-years old multiparous patient referred to Francois Quesnay Hospital for perineal and pelvic pain symptoms which had started six months after the placement of a trans-obturator tape (TOT) urethral sling for stress urinary incontinence. She presented with continuous anogenital pain, severe dyspareunia (VAS score: 10/10) and inability to stay in a sitting position. Medical treatment had failed to relieve symptoms as the patient was treated with escalating levels of pain medication without improvement. She had partial relief with osteopathic treatment, followed by the use of anticonvulsants.

In January 2015, following the aggravation of her symptoms with severe impairment of her quality of life, the patient was referred to a specialist in neuropathic pain, and a diagnostic right pudendal nerve block was performed with successful achievement of pain relief for 7 hours. The patient underwent three cycles of therapeutic transvaginal pudendal nerve block using the combination of lidocaine and corticosteroids, as per Case 1, allowing symptom improvement for 10 to 12 weeks each time.

Pelvis evaluation with Magnetic Resonance Imaging showed normal findings and no signs of endometriosis.

**Figure 3:**
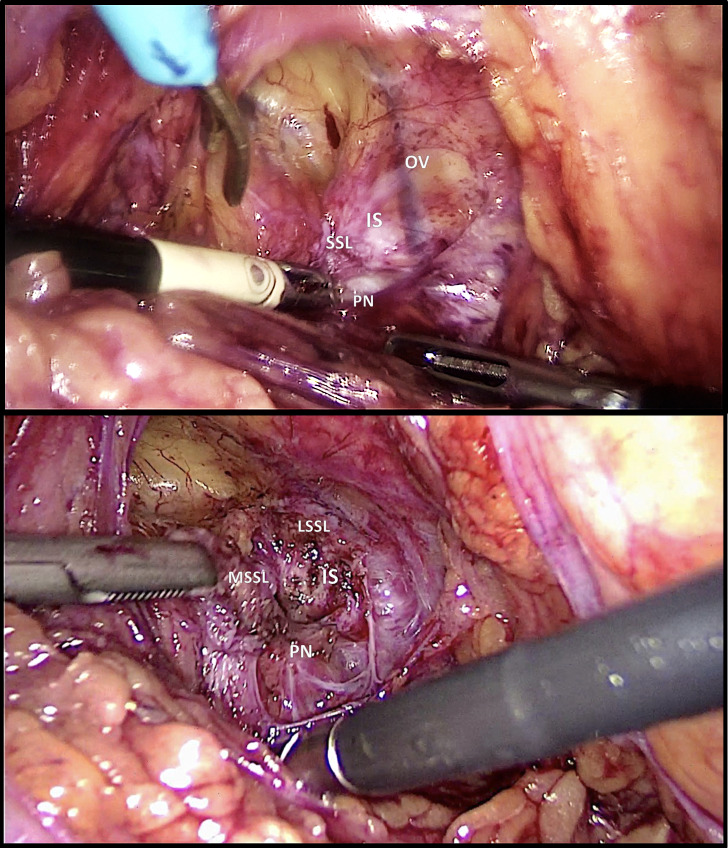
Case 2 laparoscopy. The sacrospinous ligament is retracted by the TOT arm (above) and is then transected (below). Ischial spine IS; Sacrospinous ligament SSL: medial part MSSL, lateral part LSSL; Obturator vein OV; Pudendal nerve PN.

A vaginal TOT removal combined with surgical pudendal neurolysis with a transgluteal approach was recommended but was refused by the patient. After further aggravation of her symptomatology, she was referred by her physiotherapist to Francois Quesnay Hospital, where a laparoscopic resection of the TOT with pudendal neurolysis was suggested and desired by the patient. The surgery followed the classical steps described above. The arm of the TOT sling pierced the obturator muscle posteriorly causing severe local fibrosis and retraction of the sacrospinouns ligament at the level of the ischial spine. The arm of the TOT sling was cut at the level of the obturator foramen, followed by a section of the sacrospinous ligament at the level of the ischial spine, allowing the complete release of the pudendal nerve (Figure 3). Anti-adhesion barrier gel was applied on the nerve area to prevent the recurrence of fibrosis.

No complications occurred during or after the procedure and the patient was discharged the day after surgery. The patient reported 50% improvement of her symptoms immediately after surgery and was managed with pelvic floor physiotherapy and oral gabapentin for 3 months. The improvement was stable at 3 and 6 months after the surgery. Continuation of the pelvic floor physiotherapy was suggested but medical therapy was stopped. Her anogenital pain was reported as 2/10 and the dyspareunia as 4/10 using VAS score. No urinary incontinence was reported.

## Discussion

PN is a rare condition of chronic neuropathic pain, usually unilateral, along the territory of the pudendal nerve often leading to impairment in quality of life. It is most often related to the entrapment of the pudendal nerve between the sacrospinous ligament and the sacrotuberous ligament. The literature regarding pudendal nerve injuries after vaginal mesh insertion is limited to few case reports, and the deviation from the classical surgical procedure due to technical errors can lead to nerve damages (Corona et al., 2008; Fisher and Lotze, 2011; Masata et al., 2012; Sancak et al., 2016). In our second clinical case, an irritation of the nerve might have occurred due to the fibrosis generated by the posteriorly deviated passage of the sling in the obturator foramen, close to the sacrospinous ligament. Five cases of pudendal neuropathy after TOT insertion were reported by Paulson, who described a three steps method for the diagnosis (Paulson and Baker, 2011). The first step is to determine the symptoms, the second is to define the neuropathic territory and the last is to perform a nerve block. The immediate relief of the symptoms and their recurrence shortly after are very helpful for the diagnosis of the pudendal neuropathy. Nantes criteria published by Labat et al. (2008) are a useful guiding tool for the diagnosis. In our two cases, the diagnosis of PN was achieved according to Nantes criteria due to the rapid recurrence of the symptoms after the nerve block and the necessity to repeat it (Labat et al., 2008).

To avoid surgery in the first instance, nerve block can be achieved with botox injections or through high- or low-frequency biphasic electrical stimulation of the pudendal nerve (Shapiro et al., 2021). By doing so, the creation of fibrotic tissue that can occur after surgery is avoided. However, symptoms often recur, and surgery can be the only chance to improve them.

The surgical decompression in the first case and the laparoscopic mesh resection in the second led to symptoms improvement and no fallout at 6 months’ follow-up for both cases. Anti-adhesive gel was used around the nerve to avoid fibrosis formation and recompression. The pudendal neurolysis has been described in many different approaches: transgluteal, which is the classical and the most invasive one described by Robert et al. (1993); transperineal; transischiorectal, described by Shafik; laparoscopic described by Possover (2009); or robotic approach described by Moscatiello et al. (2018). The laparoscopic approach allows a better vision, a precise dissection and is able to rule out other diseases in the pelvis. Nonetheless, it requires a developed anatomical expertise and has an extensive learning curve (Sheng et al., 2017).

Both of our cases expressed improvement of symptoms decreasing pain VAS scores of around 50%, which is defined as a success of the technique in the literature.

The residual pain can be explained by the ischemic changes of the neurons after a chronic compression, which often do not regress completely. Therefore, it is mandatory to diagnose and manage the neuropathy as early as possible; the later the decompression is performed, the worse are the results of the surgery. Another possible component of the residual pain is psychological since patients with chronic pain syndromes often develop a frustration and depression after recurrent failed treatment (Rayner et al., 2016; Sheng et al., 2017).

## Conclusion

PN is a rare condition and can be a consequence of pudendal nerve entrapment or pelvic floor surgery, especially after mesh insertion. The diagnosis and the management should not be delayed because this is the main prognostic factor of the therapy. The treatment should be individualised and should be carried out by a multidisciplinary team including a physiotherapist, anaesthesiologist, pain specialist, radiologist, and expert surgeon. When conservative treatment fails, and symptoms recur after nerve block, laparoscopic nerve exploration and decompression is an adequate option to propose and should be performed by a trained surgeon.
